# Song discrimination by nestling collared flycatchers during early development

**DOI:** 10.1098/rsbl.2016.0234

**Published:** 2016-07

**Authors:** S. Eryn McFarlane, Axel Söderberg, David Wheatcroft, Anna Qvarnström

**Affiliations:** Animal Ecology, Evolutionary Biology Centre, Uppsala University, Norbyvägen 18D, 753 26 Uppsala, Sweden

**Keywords:** species recognition, song, metabolic rate, *Ficedula* flycatcher

## Abstract

Pre-zygotic isolation is often maintained by species-specific signals and preferences. However, in species where signals are learnt, as in songbirds, learning errors can lead to costly hybridization. Song discrimination expressed during early developmental stages may ensure selective learning later in life but can be difficult to demonstrate before behavioural responses are obvious. Here, we use a novel method, measuring changes in metabolic rate, to detect song perception and discrimination in collared flycatcher embryos and nestlings. We found that nestlings as early as 7 days old respond to song with increased metabolic rate, and, by 9 days old, have increased metabolic rate when listening to conspecific when compared with heterospecific song. This early discrimination between songs probably leads to fewer heterospecific matings, and thus higher fitness of collared flycatchers living in sympatry with closely related species.

## 1. Introduction

When males produce signals that are only preferred by conspecific females, costly heterospecific matings can be avoided. The association between male signals and female preferences can break down if both traits are learned prior to sexual maturity, and there is a risk of learning or preferring a heterospecific signal [1]. For example, both male and female songbirds imprint on the songs of adult male tutors as juveniles, which guides subsequent song learning [2]. The ability of juvenile songbirds to discriminate between conspecific and heterospecific songs is thought to ensure that songbirds learn to produce and prefer conspecific songs [3] and therefore form conspecific pairs.

The age at which nestlings discriminate among songs may have important consequences for song learning, because perception of vocalizations can be influenced by early auditory experience [4], even during embryonic stages [5,6]. If song discrimination arises only after song learning then earlier exposure to sounds, including those of heterospecifics, during this critical time may lead to inappropriate learning [7]. Previous behavioural assays have shown that song discrimination can arise by the time birds have fledged the nest [8–11]. However, we do not know whether song discrimination arises even earlier, prior to the expression of observable behavioural responses.

The auditory brain regions underlying song discrimination differentiate even before hatching [12], suggesting that neural responses to song may be present well before behavioural responses. The brain is a metabolically costly organ, suggesting that physiological responses to songs, such as increased heart rate, could be used to evaluate discrimination of acoustic cues at very young and even embryonic developmental stages, as suggested by Shizuka [10]. For example, heart rate responses have been used to evaluate discrimination in fledgling songbirds [8] and even in embryos of precocial species [13]. Here, we evaluate song discrimination ability in embryonic and nestling songbirds, before individuals are capable of producing visible or audible behavioural responses, using metabolic rate as a proxy for physiological and neural responses to different song types.

Collared flycatchers (*Ficedula albicollis*) are cavity-nesting passerines that co-occur with closely related pied flycatchers (*F. hypoleuca*) in central Europe and on the Baltic island of Öland [14]. While pied flycatcher males often learn and incorporate song elements from collared flycatchers, leading to mixed song, collared flycatcher males produce only pure collared flycatcher songs [15,16]. This difference suggests that collared flycatchers have particularly selective song discrimination that arises before song learning. Collared flycatchers express strong behavioural discrimination of conspecific songs as 12-day-old nestlings, but it is unknown when collared flycatchers first express song discrimination [17]. Here, we used metabolic rate to determine whether collared flycatcher nestlings express physiological responses related to discrimination of songs prior to behavioural responses. We assumed that an increase in metabolic rate when exposed to song following a silence treatment was indicative of neural responses, and we therefore predicted that nestlings that were discriminating between songs would have higher metabolic responses to conspecific when compared with heterospecific songs.

## 2. Material and methods

We have been monitoring populations of collared and pied flycatchers breeding in nest boxes on the Swedish island of Öland (57°10′ N, 16°56′ E) since 2002. By monitoring nest boxes at regular intervals, we determined the precise laying date, hatching date, and thus age of all nestlings. Both parents were ringed and measured, females during incubation and males while feeding nestlings. Collared and pied flycatchers sometimes hybridize [14], but we did not include any nestling that had mixed-species parents in our experiment. All nestlings were ringed with unique alphanumeric rings, weighed and had blood sampled at 6 days post-hatching and were re-weighed at 12 days post-hatching.

**Figure 1.**
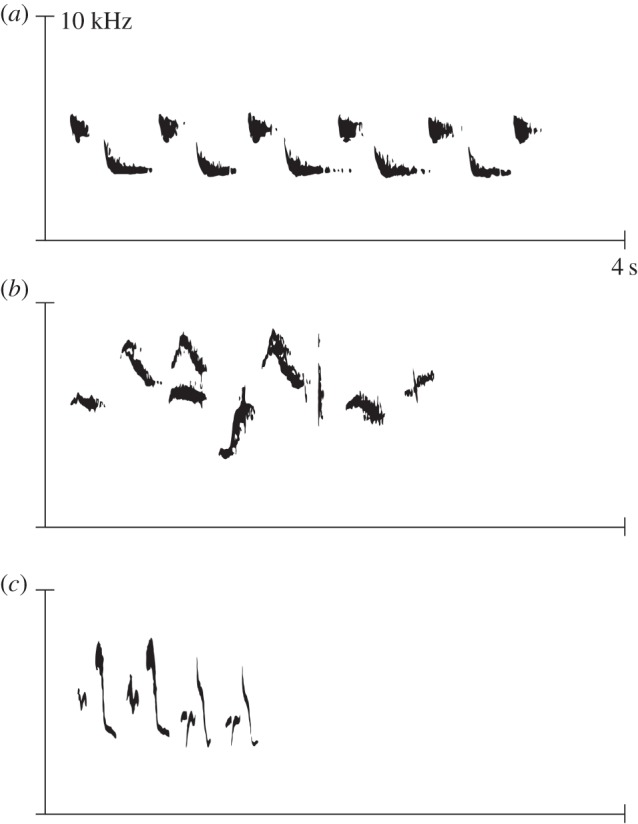
Spectrograms (frequency by time plots) of typical songs from (*a*) great tit, (*b*) collared flycatcher and (*c*) pied flycatcher.

To test whether collared flycatcher embryos and hatched nestlings had different metabolic responses to conspecific and heterospecific songs, we measured the change in respiration during the period prior to and during song playbacks, hereafter referred to as ‘metabolic response’. We used a respirometer, where embryos and nestlings were kept at a constant temperature, and air parameters were measured once per second, although we used the mean oxygen estimate over each period as our response (see the electronic supplementary material for more details). In 2015, we collected eggs 2 days prior to the expected hatching date, as well as 4-, 7-, 9- and 12-day-old nestlings. Eggs and nestlings were exposed to 5 min of silence followed by 5 min of song from collared flycatchers or from heterospecific birds, specifically pied flycatchers and great tits (figure 1). We completed two consecutive trials with different song playbacks, but discarded the second trial due to order effects, following [10] (electronic supplementary material).

We used linear mixed effects models to test whether the metabolic response could be explained by the age of the nestling, the type of song played (either great tit, pied flycatcher or collared flycatcher), or an interaction between nestling age and song type. Additionally, we included the nest identity as a random effect to control for a shared environment. The focal age response was always compared to the embryonic response, and metabolic responses to heterospecific songs were always compared to the metabolic response to conspecific song. We implemented all models in lme4 in R [18,19] and used Satterthwaite approximation in lmerTest [20] to determine degrees of freedom and assess significance.

## 3. Results

We measured 15 embryos and 45 nestlings (17 four-day-old nestlings, 11 seven-day-old nestlings, 9 nine-day-old nestlings and 8 twelve-day-old nestlings) in 2015 (the specific sample sizes are in electronic supplementary material, table S1). We found that nestlings have an increased metabolic rate response to sound (i.e. either great tit, collared or pied flycatcher song) compared with a silence treatment as they age, where 7-day-old (est = 0.153 ± 0.04, *t*_55_ = 4.03, *p* = 0.0002), 9-day-old (est = 0.173 ± 0.04, *t*_54_ = 4.29, *p* = 7.61 × 10^−05^) and 12-day-old (est = 0.136 ± 0.04, *t*_54_ = 3.25, *p* = 0.002) nestlings responded more than embryos in eggs did, while 4-day-old nestlings responded similarly to embryos (electronic supplementary material, table S2).

**Figure 2.**
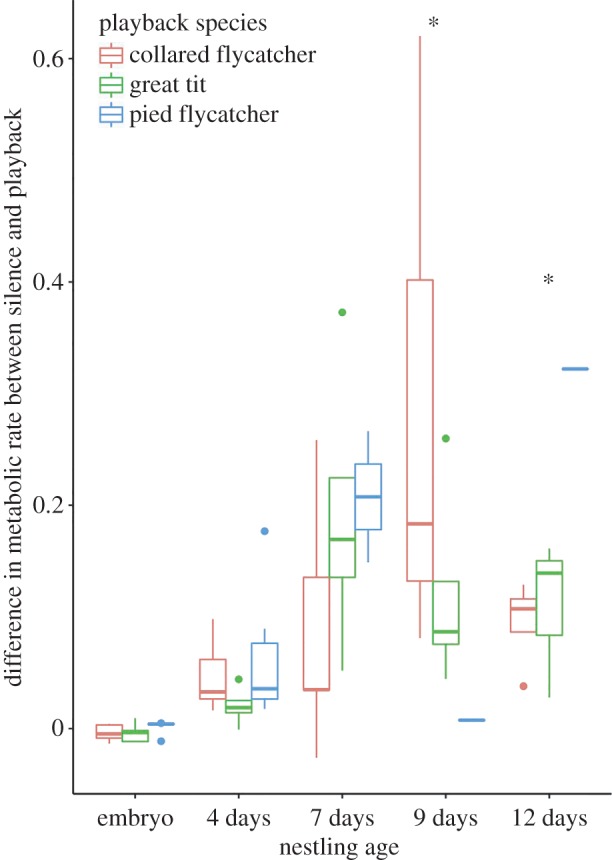
The differences in metabolic response of collared flycatchers at five different ages when played conspecific and two different heterospecific songs, displayed as a Tukey's boxplot. The asterisks denote a significant interaction.

We further investigated whether nestlings had differential responses to conspecific and heterospecific songs by comparing the increase in metabolic rate to different song treatments. We found that nestling responses depended on the song type they were exposed to. Seven-day-old nestlings did not respond differently to the various song types (figure 2; electronic supplementary material, table S3). However, 9-day-old nestlings had a higher metabolic response to conspecific collared song than to the heterospecific song playbacks, while 12-day-old nestlings responded more strongly to heterospecific pied flycatcher song than to collared song (figure 2; electronic supplementary material, table S3).

## 4. Discussion

We demonstrate that collared flycatcher nestlings had increased metabolic rates in response to song playbacks 7 days after hatching, more than 10 days before fledging. As we did not use a non-song playback as a control, we can only conclude that they can respond to sound at this age (and not specifically to song). More importantly, we found that as young as 9-day-old collared flycatcher nestlings had a higher metabolic response to collared flycatcher song than to either pied flycatcher or great tit song. While 12-day-old nestlings responded greatest to pied flycatcher song, the small sample size of pied flycatcher song playback (*n* = 1 at 9 days, *n* = 1 at 12 days; electronic supplementary material, table S1), limits our ability to compare different heterospecific song types specifically.

Our results suggest that collared flycatchers begin to respond to sounds as early as 7 days post-hatching, and are able to discriminate between conspecific and heterospecific songs as early as 9 days post-hatching. The regions in the brain involved in song processing, discrimination and learning differentiate and become interconnected at different time periods in development [12]. For example, the thalamic auditory regions, involved in sound discrimination, begin to develop before hatching; the auditory cortex, which selectively responds to conspecific song (reviewed in [21]), differentiates only after hatching; and connections between the auditory cortex and song system, important for song learning and production, arise closer to the date of fledging [12]. This staggered, but rapid development of the songbird auditory system could explain why nestlings first demonstrate metabolic rate responses to all songs at day 7, but then begin to discriminate at day 9 (figure 2).

In contrast to 9-day-old nestlings, 12-day-old collared flycatcher nestlings had a higher metabolic response to heterospecific songs than to collared flycatcher songs (figure 2). This apparent reversal of song discrimination ability may be because 12-day-old collared flycatcher nestlings produce a wide-variety of behaviours in response to sounds. In response to collared flycatcher songs, they generally jump and beg, while, in response to alarm calls and pied flycatcher songs, they duck and freeze ([22]; electronic supplementary material, video S1). When hearing pied flycatcher or great tit songs, collared flycatcher nestlings may increase vigilance in expectation of alarm, which is likely to be associated with increased metabolic response. By contrast, 9-day-old nestlings show little behavioural responses to songs or alarm calls [17], suggesting that the metabolic rate response observed at this stage is a more straightforward indicator of song discrimination.

When song discrimination arises before song learning, juveniles may be in less danger of misimprinting and ultimately producing or preferring heterospecific songs. In most songbirds, song imprinting and learning is thought to occur after juveniles fledge from the nest [2]. The ability of collared flycatchers to discriminate songs well before the song-learning period [23] may explain why they rarely produce mixed-species song [16]. Because collared flycatchers co-occur with pied flycatchers throughout most of their breeding range, selection may have favoured early song discrimination to avoid production of mixed-species songs [15,16], that may attract hetersopecific females [24]. By contrast, pied flycatchers have a larger, and more northern breeding range making it likely that pied flycatchers breeding in the young hybrid zone on Öland lack recent historical exposure to collared flycatchers, which could preclude selection for song discrimination, and subsequently result in increased mixed singing when the species are in sympatry [16]. Future work could determine whether pied flycatchers in this system lack the ability to discriminate early in the nestling phase, leading to their apparent lack of discrimination as fledglings [17] and common production of mixed-species song as adults [16].

Passerine nestlings develop consistently exposed to a cacophony of sounds that they must ignore in order to learn their conspecific song adequately. By developing song discrimination well before song learning, as appears to be the case in collared flycatchers and other species [10], nestlings may be at less risk for mistaken mate choice when they become sexually mature [25]. Establishing the timing of song recognition and discrimination allows us to identify the stages in nestling brain development that are likely to be under strong selection to prevent hybridization later in life, and give further insight into pre-zygotic isolation in wild systems.

## Supplementary Material

Supplementary Material

## Data accessibility

The data for this project are available in Dryad http://dx.doi.org/10.5061/dryad.8n45g [26].

## References

[1] Verzijden MN, Ten Cate C, Servedio MR, Kozak GM, Boughman JW, Svensson EI 2012 The impact of learning on sexual selection and speciation. Trends Ecol. Evol. 27, 511–519. (10.1016/j.tree.2012.05.007) 22705159

[2] Bolhuis JJ, Moorman S 2015 Birdsong memory and the brain: in search of the template. Neurosci. Biobehav. Rev. 50, 41–55. (10.1016/j.neubiorev.2014.11.019) 25459663

[3] Marler P 1997 Three models of song learning: evidence from behavior. J. Neurobiol. 33, 501–516. (10.1002/(SICI)1097-4695(19971105)33:5<501::AID-NEU2>3.0.CO;2-8) 9369456

[4] Woolley S 2012 Early experience shapes vocal neural coding and perception in songbirds. Dev. Psychobiol. 54, 612–631. (10.1002/dev.21014) 22711657 PMC3404257

[5] Harshaw C, Lickliter R 2011 Biased embryos: prenatal experience alters the postnatal malleability of auditory preferences in bobwhite quail. Dev. Psychobiol. 53, 291–302. (10.1002/dev.20521) 21400491

[6] Colombelli-Négrel D, Hauber ME, Robertson J, Sulloway FJ, Hoi H, Griggio M, Kleindorfer S 2012 Embryonic learning of vocal passwords in superb fairy-wrens reveals intruder cuckoo nestlings. Curr. Biol. 22, 2155–2160. (10.1016/j.cub.2012.09.025) 23142041

[7] Grant BR, Grant PR 1996 Cultural inheritance of song and its role in the evolution of Darwin's finches. Evolution 50, 2471–2487. (10.2307/2410714) 28565664

[8] Dooling R, Searcy M 1980 Early perceptual selectivity in the swamp sparrow. Dev. Psychobiol. 13, 499–506. (10.1002/dev.420130508) 7409330

[9] Nelson DA, Marler P 1993 Innate recognition of song in white-crowned sparrows: a role in selective vocal learning? Anim. Behav. 46, 806–808. (10.1006/anbe.1993.1258)

[10] Shizuka D 2014 Early song discrimination by nestling sparrows in the wild. Anim. Behav. 92, 19–24. (10.1016/j.anbehav.2014.03.021)

[11] Braaten RF, Reynolds K 1999 Auditory preference for conspecific song in isolation-reared zebra finches. Anim. Behav. 58, 105–111. (10.1006/anbe.1999.1134) 10413546

[12] Kirn JR 2010 The relationship of neurogenesis and growth of brain regions to song learning. Brain Lang. 115, 29–44. (10.1016/j.bandl.2009.09.006) 19853905 PMC2888937

[13] Gottlieb G 1979 Development of species identification in ducklings: V. Perceptual differentiation in the embryo. J. Comp. Physiol. Psychol. 93, 831 (10.1037/h0077614)

[14] Qvarnström A, Rice AM, Ellegren H 2010 Speciation in *Ficedula* flycatchers. Phil. Trans. R. Soc. B 365, 1841–1852. (10.1098/rstb.2009.0306) 20439285 PMC2871891

[15] Lundberg A, Alatalo RV 1992 The pied flycatcher. London, UK: A&C Black.

[16] Haavie J, Borge T, Bures S, Garamszegi LZ, Lampe H, Moreno J, Qvarnström A, Török J, Sætre GP 2004 Flycatcher song in allopatry and sympatry—convergence, divergence and reinforcement. J. Evol. Biol. 17, 227–237. (10.1111/j.1420-9101.2003.00682.x) 15009256

[17] Wheatcroft D, Qvarnström A Submitted. Innate song discrimination in a closely related songbird pair.

[18] Bates D, Maechler M, Bolker B, Walker S 2014 lme4: Linear mixed-effects models using Eigen and S4. R package version 1.1-7. See http://CRAN.R-project.org/package=lme4.

[19] R Core Team. 2013 R: a language and environment for statistical computing. Vienna, Austria: R Foundation for Statistical Computing (http://www.R-project.org/)

[20] Kuznetsova A, Brockhoff PB, Christensen RHB 2014 lmerTest: tests for random and fixed effects for linear mixed effects models. See https://CRAN.R-project.org/package=lmerTest.

[21] Wheatcroft D, Qvarnström A 2015 A blueprint for vocal learning: auditory predispositions from brains to genomes. Biol. Lett. 11, 20150155 (10.1098/rsbl.2015.0155) 26246333 PMC4571673

[22] Wheatcroft D 2015 Repetition rate of calls used in multiple contexts communicates presence of predators to nestlings and adult birds. Anim. Behav. 103, 35–44. (10.1016/j.anbehav.2015.02.009)

[23] Espmark YO, Lampe HM 1993 Variations in the song of the pied flycatcher within and between breeding seasons. Bioacoustics 5, 33–65. (10.1080/09524622.1993.9753229)

[24] Ålund M, Immler S, Rice AM, Qvarnström A 2013 Low fertility of wild hybrid male flycatchers despite recent divergence. Biol. Lett. 9, 20130169 (10.1098/rsbl.2013.0169) 23576780 PMC3645050

[25] Ritchie MG 2007 Sexual selection and speciation. Annu. Rev. Ecol. Evol. Syst. 37, 79–102. (10.1146/annurev.ecolsys.38.091206.095733)

[26] McFarlane SE, Söderberg A, Wheatcroft D, Qvarnström A 2016 Data from: Song discrimination by nestling collared flycatchers during early development. Dryad Digital Repository. (10.5061/dryad.8n45g) PMC4971166 27405379

