# Supplementary material for: Song discrimination by nestling collared flycatchers during early development
Source: Biol Lett. 2016 Jul;12(7):20160234. doi: 10.1098/rsbl.2016.0234 (PMC4971166; doi:10.1098/rsbl.2016.0234)
Supplement: Supplementary Material [file rsbl20160234supp1.docx]

**Supplementary Material for “Song Discrimination by nestling collared flycatchers during early development”.**

S. Eryn McFarlane^1^*, Axel Söderberg^1^, David Wheatcroft^1^, and Anna Qvarnström^1^

^1^Animal Ecology, Evolutionary Biology Centre, Uppsala University, Norbyvägen 18D, 753 26 Uppsala, Sweden

*Corresponding author

**Song recordings:**

All flycatcher songs were recorded on Öland using DAT and minidisc recorders (Sony TCD-D7, TCD-D10 and Kenwood DMC-J7R), with microphones that were mounted on 55cm parabolic dishes (Classic, Science and PRO II, Telinga, Tobo, Sweden). Great tit songs were high-quality mp3 recordings obtained from an open-access online database of avian sounds (www.xeno-canto.org; accession numbers: XC237310, XC156084, XC235098, XC218105, XC211493, XC211492, XC200096, XC183203, XC212210, XC212209). All but one recording (with three unique songs) contained at least five unique songs. All recordings were edited of background noise, low bandpass filtered at 1 kHz, and each song was standardized to a similar amplitude using RAVEN Pro v.1.4 [1]. Available from http://www.birds.cornell.edu/raven**.** Five minute playback recordings were created by repeatedly copying and pasting the sequence of unique songs. Song sequences were kept at their natural repetition rate (i.e., songs per minute), but were similar across species (pied: 10.80 songs/minute ± 0.66 SE, collared: 9.40 ± 0.43, great tit ± 0.75).

**Song playbacks:**

Songs were played back through an mp3 player (iPod nano, Apple Corp, USA) attached to a portable speaker (Grace Digital AQCSE101 Eco Extreme Portable Case). Each experiment consisted of two playback trials, using song recordings of two of the three species. The combination of songs and the order in which they were played back were alternated in successive trials.

Nestling and Embryo Sampling:

We measured two eggs from each nest for embryo trials, and nestlings were measured alone. We tended to not use individuals from the same nests for trials of different ages to avoid sampling of the same individual twice. However, we cannot be certain that we did not re-sample the same individual from the same nest when nestlings or eggs that we too young to be ring marked. We have accounted for repeated nest sampling statistically (see Statistical Methods).

**Respirometry:**

We measured either two eggs together or one nestling at the same time using a respirometer. The metabolic rate of eggs have been previously measured in respirometer in other studies [2, 3], and we saw a noted increased in oxygen consumption of chambers that included eggs compared to empty, baseline chambers, indicating that it is possible to measure embryonic metabolic rate. Nestlings were kept in a climate cabinet at 28°C to keep them in their thermoneutral zone while being measured [4], while eggs were kept at 33°C to mimic the average temperature in a nest during incubation (P. Sirkiä pers. comm). We used a FMS respirometer together with a RM-8 multiplexer, PP-2H field pump and a FlowBar-8 (Sable Systems, Henderson, NV, USA). We calibrated the respirometer using ascarite and dririte for the oxygen monitor, a standard carbon dioxide concentration in nitrogen gas for the carbon dioxide monitor and magnesium perchlorate for water vapor pressure as suggested by the manufacturer. We used approximately 50 ml/minute of air for our egg trials, 200 ml/minute of air for our four-day-old nestlings and 400 ml/minute of air for nestlings that were seven, nine and twelve days old, to account for differences in mass. Air samples were taken every second, and the mean estimate for rate of oxygen consumption (i.e. VO2) over this time was used metabolic response.

**Second cycle of playbacks:**

We ran two cycles of playbacks on each nestling and pair of eggs. We always began with one period of 5 minutes of silence, followed by a playback of songs from one species, five more minutes of silence and a playback of songs from a different species. In our results, we report only the first cycle of each trial because the metabolic rate in second trial was highly correlated with the first trial (cor= 0.975, CI= 0.96 – 0.98, t_58_=33.186, p<2.2e-16). This was driven by an elevated metabolic rate throughout the second rest period, compared to the first rest period (t_59_=5.33, p=1.6e-06), suggesting that nestlings and embryos did not settle back to baseline metabolism between trials.

To account for a possible acclimatization period that embryos or nestlings might have needed when first put in the respirometry chambers in the climate cabinet (i.e. time they might have needed to enter their thermoneutral zone), we ran additional models that included the slope of oxygen consumption over the five minute silence treatment as an additional fixed factor. We found no significant effect of this on the metabolic response of nestlings in this study (Supplementary Table 4).

**Supplementary Videos:**

Video 1) Video recording of an entire clutch of twelve-day-old collared flycatcher nestlings showing typical responses to songs of collared flycatchers. Nestlings look up at the nest box opening, produce begging calls, and display begging gapes.

Video 2) Video recording of the same clutch of twelve-day-old collared nestlings showing typical responses to songs of pied flycatchers. Nestlings move initially, but thereafter crouch and most remain motionless for the duration of the playback.

**Supplementary Table 1:** Number of individuals tested in each age – song treatment in 2015.

| Playback Species | Embryo | Four-day-old | seven-day old | nine-day old | twelve-day-old |
| --- | --- | --- | --- | --- | --- |
| Collared flycatcher | 5 | 5 | 5 | 3 | 4 |
| Great tit | 5 | 6 | 4 | 5 | 3 |
| Pied flycatcher | 5 | 6 | 2 | 1 | 1 |

**Supplementary Table 2:** A linear mixed effects model in which metabolic response is explained by nestling age where a nest effect is the random effect. The contrast for all fixed effects is the response to song exhibited by embryos two days prior to hatching; thus, a significant term is significantly different from the response by embryos to a song playback. We report the beta estimate, standard error (Std error), degree of freedom (df), t value and p value for each fixed effect, as well as the variance and standard deviation (Std. dev) for the random effect.

| Fixed Effects | Estimate | Std error | df | t value | p value |
| --- | --- | --- | --- | --- | --- |
| Intercept | -0.005 | 0.03 | 55 | -0.02 | 0.841 |
| 4 days old | 0.047 | 0.03 | 53.9 | 1.39 | 0.170 |
| **7 days old** | **0.153** | **0.04** | **54.9** | **4.03** | **0.000** |
| **9 days old** | **0.173** | **0.04** | **54.1** | **4.28** | **0.000** |
| **12 days old** | **0.136** | **0.04** | **54.1** | **3.25** | **0.002** |
| Random Effects | Variance | Std Dev |  |  |  |
| Nestbox | 0.0009 | 0.03 |  |  |  |
| Residual | 0.00083 | 0.091 |  |  |  |

**Supplementary Table 3**: A second linear mixed effects model as in Table 1, with the same random effects and an interaction between age and the species of song played to the nestling, where the response variable is the difference in metabolic rate between when a nestling was exposed to silence and a song playback. The base contrast for the fixed effect of age is the response by embryos two days prior to hatching, and the base contrast for the fixed effect of song is the response to collared flycatcher song. Interactions can be interpreted as the difference in metabolic response, defined as the difference in metabolic rate between listening to song and listening to silence, between nestlings of the age in the table (i.e. 4, 7, 9 or 12 days) and embryos when listening to heterospecific song (pied flycatcher or great tit) compared to collared flycatcher songs. For example, the difference in response between 9-day-old nestlings and embryos is larger when listening to collared song than when listening to either great tit song or pied flycatcher song. We report the beta estimate, standard error (Std error), degree of freedom (df), t value and p value for each fixed effect, as well as the variance and standard deviation (Std. dev) for the random effect.

| Fixed Effects | Estimate | Std error | df | t value | p value |
| --- | --- | --- | --- | --- | --- |
| intercept | -0.003 | 0.04 | 44.75 | -0.080 | 0.936 |
| 4 days old | 0.033 | 0.05 | 44.54 | 0.598 | 0.553 |
| 7 days old | 0.101 | 0.05 | 43.91 | 1.856 | 0.070 |
| **9 days old** | **0.309** | **0.06** | **43.91** | **4.932** | **0.000** |
| 12 days old | 0.106 | 0.06 | 44.55 | 1.827 | 0.074 |
| Great tit song | -0.001 | 0.05 | 45.00 | -0.013 | 0.990 |
| Pied flycatcher song | -0.010 | 0.05 | 44.60 | -0.177 | 0.860 |
| 4 days old x Great tit song | -0.009 | 0.08 | 45.00 | -0.113 | 0.911 |
| 7 days old x Great tit song | 0.095 | 0.08 | 44.68 | 1.194 | 0.239 |
| **9 days old x Great tit song** | **-0.186** | **0.08** | **44.71** | **-2.230** | **0.030** |
| 12 days old x Great tit song | 0.009 | 0.09 | 44.42 | 0.108 | 0.915 |
| 4 days old x Pied Flycatcher Song | 0.044 | 0.08 | 44.28 | 0.591 | 0.557 |
| 7 days old x Pied Flycatcher Song | 0.122 | 0.09 | 43.32 | 1.365 | 0.179 |
| **9 days old x Pied Flycatcher Song** | **-0.288** | **0.11** | **44.71** | **-2.513** | **0.016** |
| **12 days old x Pied Flycatcher Song** | **0.230** | **0.11** | **44.99** | **2.068** | **0.044** |
| Random Effects | Variance | Std dev |  |  |  |
| \| Nestbox \| \| --- \| | 0.002 | 0.04 |  |  |  |
| Residual | 0.006 | 0.08 |  |  |  |

**Supplementary Table 4:** A linear mixed effects model, as in Supplementary Table 3, to determine the effect of age and song species in the playback on metabolic response in nestling collared flycatchers. In addition, we have added the slope of O2 consumption during the five-minute silent treatment to account for any acclimatization that nestlings and embryos may have needed after being placed in a respirometry chamber.

| Fixed Effects | Estimate | Std error | df | t value | p value |
| --- | --- | --- | --- | --- | --- |
| intercept | -0.003 | 0.04 | 43.82 | -0.080 | 0.937 |
| Slope of O_2_ consumption | -0.037 | 0.67 | 40.05 | -0.056 | 0.9559 |
| 4 days old | 0.031 | 0.06 | 43.59 | 0.575 | 0.5684 |
| 7 days old | 0.100 | 0.06 | 42.73 | 1.821 | 0.0755 |
| **9 days old** | **0.308** | **0.07** | **41.77** | **4.696** | **2.9e-05** |
| 12 days old | 0.104 | 0.07 | 43.94 | 1.543 | 0.1300 |
| Great tit song | -0.001 | 0.06 | 43.98 | -0.012 | 0.9905 |
| Pied flycatcher song | -0.010 | 0.05 | 43.64 | -0.183 | 0.8557 |
| 4 days old x Great tit song | -0.008 | 0.08 | 44.00 | -0.105 | 0.9168 |
| 7 days old x Great tit song | 0.093 | 0.08 | 43.96 | 1.112 | 0.2722 |
| **9 days old x Great tit song** | -0.187 | 0.09 | 43.97 | -2.148 | 0.0372 |
| 12 days old x Great tit song | 0.010 | 0.09 | 43.34 | 0.113 | 0.9102 |
| 4 days old x Pied Flycatcher Song | 0.045 | 0.08 | 43.33 | 0.596 | 0.5546 |
| 7 days old x Pied Flycatcher Song | 0.121 | 0.09 | 43.38 | 1.293 | 0.2027 |
| **9 days old x Pied Flycatcher Song** | -0.289 | 0.12 | 43.40 | -2.456 | 0.0181 |
| **12 days old x Pied Flycatcher Song** | 0.225 | 0.14 | 43.44 | 1.558 | 0.1265 |
| Random Effects | Variance | Std dev |  |  |  |
| \| Nestbox \| \| --- \| | 0.002 | 0.04 |  |  |  |
| Residual | 0.006 | 0.08 |  |  |  |

[1] Program, B.R. 2011 Raven Pro: Interactive Sound Analysis Software. (ed. T.C.L.o. Ornithology). Ithaca, NY.

[2] Boonstra, T.A., Clark, M.E. & Reed, W.L. 2010 Position in the sequence of laying, embryonic metabolic rate, and consequences for hatching synchrony and offspring survival in Canada Geese. *The Condor* **112**, 304-313.

[3] Vleck, C.M., Hoyt, D.F. & Vleck, D. 1979 Metabolism of avian embryos: patterns in altricial and precocial birds. *Physiological Zoology*, 363-377.

[4] Lasiewski, R.C., Hubbard, S. & Moberly, W. 1964 Energetic relationships of a very small passerine bird. *Condor*, 212-220.
